# Networking in eHealth research: results of the IDRC SEARCH program evaluation

**DOI:** 10.1093/pubmed/fdy193

**Published:** 2018-12-14

**Authors:** Josef Decosas, Lawrence Mbuagbaw

**Affiliations:** 1 hera, Belgium; 2 Department of Health Research Methods, Evidence and Impact, McMaster University, Hamilton, Ontario, Canada

## Abstract

**Background:**

The IDRC ‘Strengthening Equity through Applied Research Capacity building in eHealth’ (SEARCH) funded seven research projects in Bangladesh, Burkina Faso, Ethiopia, Kenya, Lebanon, Peru and Vietnam that sought to answer questions or test solutions related to the use of Internet or mobile phone technology in strengthening health systems. The evaluation accompanied these projects over two years to answer, among others, the question how cross-grant learning interactions influenced project outcomes.

**Methods:**

The evaluation team conducted repeated interviews and on-line questionnaire surveys with the research teams and analysed the information exchanges among researchers on a SharePoint site established by IDRC.

**Results:**

The expectations of the SEARCH program in terms of cross-project learning were only partially realized. The diversity of themes, language barriers and differences in context were cited as main reasons. Non-facilitated active cross-grant networking was only observed between two teams working in English on thematically similar issues. However, networking among all projects was active during two program workshops organized by IDRC.

**Conclusions:**

Networking among research teams can increase the quality and the applicability of health systems research and potentially promote knowledge translation. Spontaneous networking across language barriers is, however, difficult. Effective global research networks require dedicated human and financial resources to keep them vibrant and alive.

**Keywords:**

e-health, refugees

## RESUMEN

**Contexto:**

El programa "Fortalecimiento de la equidad mediante el desarrollo de capacidad de investigación aplicada para la cibersalud (eHealth)" (SEARCH) del CRDI financió siete proyectos de investigación en Bangladesh, Burkina Faso, Etiopía, Kenia, el Líbano, Perú y Vietnam. Estos proyectos buscaban responder preguntas o probar soluciones relacionadas con el uso de Internet o tecnología de teléfonos móviles para consolidar los sistemas de salud. La evaluación acompañó a estos proyectos más de dos años para responder, entre otras cosas, la pregunta acerca de cómo las interacciones de aprendizaje a través de los proyectos subvencionados incidieron en los resultados de los proyectos.

**Métodos:**

El equipo de evaluación llevó a cabo reiteradas entrevistas y encuestas en línea con los equipos de investigación y se analizaron los intercambios de información entre los investigadores en un sitio SharePoint que estableciera el CRDI.

**Resultados:**

Las expectativas del programa SEARCH con relación al aprendizaje de proyectos transversales solo se lograron parcialmente. La diversidad de temas, barreras idiomáticas y diferencias en el contexto se citaron como razones principales. Solo se observaron redes de trabajo con subvención, activas y no facilitadas, entre dos equipos trabajando en inglés en asuntos con temas similares. No obstante, las redes de trabajo entre todos los proyectos fueron activas durante dos talleres del programa organizados por el CRDI.

**Conclusiones:**

las redes de trabajo entre los equipos de investigación pueden aumentar la calidad y la aplicabilidad de la investigación de los sistemas de salud y podrían promover la traducción del conocimiento. Sin embargo, las redes de trabajo espontáneas a través de las barreras idiomáticas son difíciles. Las redes de investigación mundial eficaces necesitan recursos financieros y personal humano dedicado para preservarlas vivas y dinámicas.

**Palabras clave:**

e-salud, m-salud, investigación, evaluación, red y redes de trabajo

## RÉSUMÉ

**Contexte:**

Le programme *Promotion de l’équité au moyen du renforcement des capacités de recherche appliquée en cybersanté* (SEARCH), du CRDI, a financé sept projets de recherche au Bangladesh, au Burkina Faso, en Éthiopie, au Kenya, au Liban, au Pérou et au Vietnam pour répondre à des questions ou mettre des solutions à l’essai pour renforcer les systèmes de santé au moyen d’Internet ou de technologies de téléphonie cellulaire. Ces projets ont fait l’objet d’une évaluation continue pendant deux ans visant notamment à comprendre l’incidence d’échanges d’information entre organismes boursiers sur les résultats des projets.

**Méthodologie:**

L’équipe évaluatrice a régulièrement interrogé les équipes de recherche, leur a transmis en ligne des enquêtes par questionnaire et a analysé les échanges d’information effectués entre chercheurs sur un site SharePoint du CRDI.

**Résultats:**

Les attentes vis-à-vis du programme SEARCH en matière d’apprentissage interprojets n’ont été que partiellement satisfaites. Cela s’explique principalement par la diversité des thèmes, l’obstacle de la langue et des contextes différents. Le seul exemple observé de réseautage actif sans médiation entre boursiers a eu lieu entre deux équipes travaillant sur des thèmes similaires en langue anglaise. Toutefois, tous les projets ont activement réseauté dans le cadre de deux ateliers du programme organisés par le CRDI.

**Conclusions:**

Le réseautage entre équipes de recherche peut bonifier la recherche sur les systèmes de santé et éventuellement, favoriser le transfert de connaissances. Cependant, le réseautage se fait rarement spontanément en présence de l’obstacle linguistique. Les réseaux de recherche internationaux exigent qu’on leur consacre des ressources humaines et financières pour assurer leur dynamisme et leur survie.

**Mots-clés:**

cybersanté, applications mobiles en santé, recherche, évaluation, réseau et réseautage

In 2012, a commentary in the journal ‘Nature’ observed a fundamental shift in the geography of science through the rise of research networks.^1^ Networks, as claimed by the author, are changing the global balance of research. An earlier meta-analysis of the citation impact of 442 publications resulting from 22 research networks found a complex relationship between the intensity of collaboration as measured by the interactions within networks, and the quality of scientific outputs as measured by the citation ratio. Networks with a high level of interaction were associated with high consistency in the quality of publications. While the quality of scientific outputs in low intensity networks was less consistent, they generated publications with both the highest and the lowest citation ratios.^2^ The findings suggest that researchers and institutions with high research capacity do not necessarily require peer networks to generate quality publications. However, in the context of research capacity building, close networking contributes to consistency in quality and lowers the risk that any research team is left behind.

Support to networking is at the core of the International Development Centre’s (IDRC) approach to funding research in developing countries. In 2004/05, IDRC carried out a strategic review of its support to networks in a series of evaluation studies looking at network coordination, governance, sustainability and development outcomes. The findings of this review informed the IDRC annual learning forum in 2005 which defined networks as ‘social arrangements of organizations and/or individuals linked together around a common theme or purpose, working jointly but allowing members to maintain their autonomy as participants’.^3^

The IDRC review of network governance, coordination and outcomes in 2005 found, not surprisingly, that networks distinguish themselves from other organizational forms by their level of participation. It also highlighted the important role of a coordinating network member to manage expectations and facilitate the direction of network activities.^4^ Research networks need investment and nurturing to reach their optimal value.

Under the IDRC SEARCH program, seven research projects were funded in Bangladesh, Burkina Faso, Ethiopia, Kenya, Lebanon, Peru and Vietnam that sought to answer questions or test solutions related to the use of Internet or mobile phone technology in strengthening health systems. The four program objectives were to (i) strengthen, redesign and improve health system processes; (ii) support key health system operations essential for equity; (iii) generate evidence for decision-making; and (iv) increase social participation, accountability and transparency. Networking for joint learning and knowledge sharing ‘to reduce research silos and to encourage dialogue and enrich discussions’ was an additional program objective.^5^

Network coordination is to some extent implicit in the IDRC approach to research funding, which includes close follow-up and mobilization of capacity support for all funded projects. The IDRC program officers thereby act as network hubs within their programs, transmitting lessons from one project to another and facilitating the exchange of information among funded projects. No additional network coordinator was appointed for SEARCH, although it was raised as an option by the participants of the SEARCH proposal development workshop.^6^

Instead, the projects funded under SEARCH were encouraged to network among themselves according to the needs and resources of each research team. At the same time, hera, a health consulting agency, was contracted to accompany the program in a learning evaluation over two years from 2014 to 2016. One of the five questions to be answered by the evaluation was: ‘How have the cross-grant learning interactions within SEARCH influenced project outcomes?’^7^ Final project outcomes could, of course, not be evaluated when the evaluation contract ended in 2016 because all seven teams were still in the process of analysing data and preparing reports. But the evaluation team monitored information exchanges among the researchers. In two rounds of interviews with the Principal Investigators in 2015 and 2016, the team explored the extent to which researchers engaged in cross-grant learning without the presence of a formally appointed network leader. The main data limitation was that networking exchanges among evaluation team members other than the Principal Investigators were not captured in the interviews. A Microsoft SharePoint site was established by IDRC to facilitate network exchanges, allowing teams to post documents, announce events such as workshops or conference presentations, and to engage in discussion fora which were, however, used sparingly.

The evaluation team assessed the patterns, types and intensity of exchanges among researchers through social network analysis. The most intensive network exchanges occurred during workshops or were directly mediated by IDRC program staff. These were removed from the analysis as they reflected the usual IDRC practice. The seven projects and the SharePoint site were retained as the eight network actors. The following metrics were calculated: Degree centrality (the total number of connections between projects); betweenness centrality (whether actors served as bridges in the shortest paths between two actors); closeness centrality (how close one actor is to another actor); and closeness (the degree to which an actor is near all other actors).^8^ The analysis was conducted using NodeXL Excel Template Version 1.0.1.342; Social Media Research Foundation.^9^

The result of the network analysis is presented in the graphic in which each of the seven projects is identified by the national flag. The proximity of the symbols indicates the number of exchanges that occurred between projects, and the opacity of the connecting line indicates the self-reported usefulness of the exchanges.

The expectations of the SEARCH program in terms of cross-project learning beyond the traditional mode of IDRC mediated exchanges and organized workshops were only partially realized. There was active networking between the two projects in Bangladesh and Kenya as can be seen by the proximity of the two icons in the graphic. These two projects were thematically similar, both working on national frameworks for eHealth policy, and the two research teams worked in English.

The project on access to health services for women, children and people living with HIV in Burkina Faso used the SharePoint site to post and retrieve materials, but as the only research team working in French, its interactions with the other teams were limited, including with the team in Vietnam which was closest in thematic terms. Language was also a constraint for the only Spanish-speaking team studying information systems in Peru. The research teams in Ethiopia and in Lebanon had relatively few interactions with the other teams or with the SharePoint site, which may be related to the subjects of their research themes which had little overlap with any other of the seven projects.


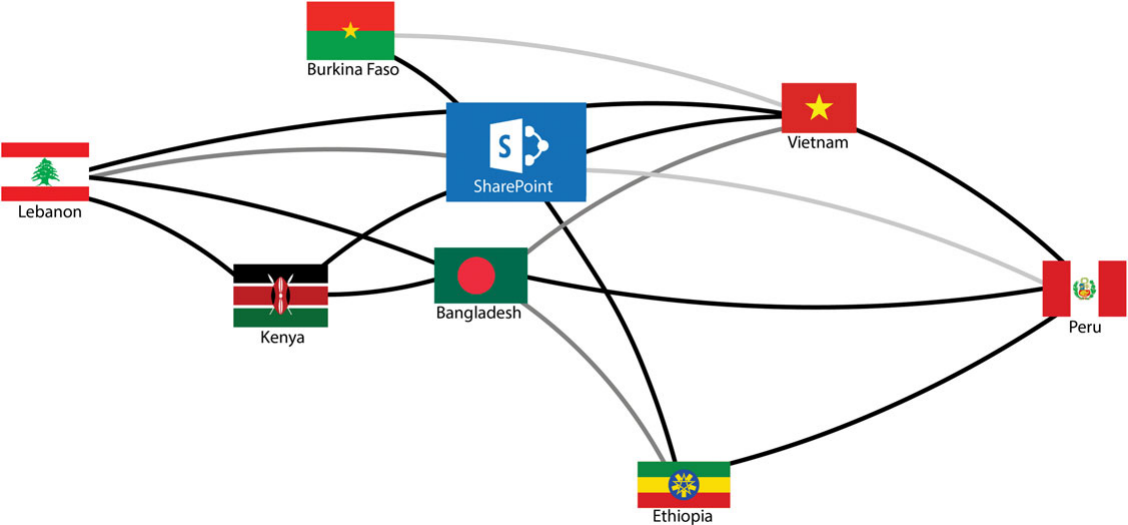


While spontaneous networking among the research teams was limited, information exchanges using the traditional modality of IDRC-managed networking were very active. The teams met and discussed common challenges and experiences during two program workshops. They participated actively in a web-based training on gender analysis. IDRC program officers used site visits and feedback on progress reports to link project outputs to overarching program objectives. In repeated survey responses, program workshops, conferences and other face-to-face interactions were consistently ranked as the preferred platforms for information exchange.

Several lessons about research networking and cross-project learning can be drawn from the SEARCH experience:

- The integration of eHealth in health systems raises many issues. There are common questions, for instance on privacy, data security and the interactivity of technological platforms that were extensively discussed in program workshops. However, the technology itself, i.e. the ‘e’ in eHealth, does not provide a sufficiently common platform for information exchange that is useful for all. When there are commonalities in the research themes, as for instance between the studies in Kenya and in Bangladesh, the research teams will use opportunities to spontaneously exchange information.
- Networking among research teams can increase the quality and the applicability of health systems research and potentially promote knowledge translation. But networking has a cost. It may happen spontaneously when a platform is provided, but it is much more active and potentially useful when it is supported by dedicated human and financial resources to keep the network alive. All seven research teams wanted to exchange information, but they wanted organized venues such as workshops or webinars.
- Spontaneous networking across language barriers is difficult. The barriers can be overcome by a network manager such as IDRC in the case of the interactions between the francophone, hispanophone and anglophone research teams in SEARCH workshops and webinars. But it is not evident that it happens spontaneously without such facilitation.
- The evaluation ended in 2016, too early to assess the quality and the impact of research conducted in the SEARCH network. Although not formally assessed, there is emerging evidence in 2018 that research conducted under the SEARCH grants left a footprint in eHealth research that was arguably enhanced by networking among the seven teams. In total, 12 research papers have already been published in peer-reviewed journals with more currently under review. In 2016 the project in Burkina Faso, and in 2017 the project in Vietnam received awards from the Fondation Pierre Fabre’s Global South e-Health Observatory; and eHealth solutions researched and piloted by SEARCH projects in Peru and Vietnam were adopted and scaled up in national or provincial health systems.

## Funding

This work was made possible by a grant from the International Development Research Centre.
